# Assessing perceived and functional health literacy among Cypriot parents

**DOI:** 10.1093/eurpub/ckac131.320

**Published:** 2022-10-25

**Authors:** I Menikou, N Middleton, E Papastavrou, C Nicolaou

**Affiliations:** Nursing, Cyprus University of Technology, Limassol, Cyprus

## Abstract

**Background:**

Health literacy (HL) refers to people’s competencies in accessing, understanding, assessing and applying health information to meet demands in healthcare, preventing illness and promoting health. In the case of children, parental HL is important in establishing health-promoting behaviours and better health outcomes.

**Methods:**

A cross-sectional study was conducted among parents of children, aged 6 months to 15 years, in Cyprus with the aim of assessing perceived and functional HL. A convenience sample of parents presenting in pediatric outpatient departments in three Cypriot cities rated their HL using the HLS-EU-Q47 and the NVS (Newest Vital Sign), a performance-based measure of HL. Participants were classified according to the overall and domain-specific scores and associations with socio-demographics were explored.

**Results:**

HLS-EU-Q47 mean score among 416 parents (83.2% female, 83.8% tertiary education) was 35.30 (SD = 7.45). Based on suggested ranges, almost half (42.6%) were classified as having inadequate or problematic HL. Consistently, 62.8% showed high likelihood or significant possibility of limited functional HL, based on the NVS with a mean score of 2.73 (SD = 2.02). Competency of understanding health information was rated higher (37.71, SD = 7.39), whereas assessing health information was rated lower (33.55, SD = 9.05). Among the three domains of the HLS-EU-Q47, the highest mean score was for healthcare (36.16, SD = 7.04) and the lowest score for health promotion (34.60, SD = 8.88). Parental HL was statistically significantly associated with education and financial difficulties.

**Conclusions:**

Moderate-to-low levels of perceived HL appear consistent with a performance-based measure of HL. As a high number of parents may face challenges in assessing and applying health information to improve outcomes for their children, healthcare services should be oriented towards identifying problematic HL, while Public Health interventions are needed to enhance parental HL.

**Key messages:**

• According to both a perceived and a performance-based measure of health literacy, the study suggests that a high proportion of parents in Cyprus may have inadequate or problematic health literacy.

• Health education interventions within clinical settings as well as at Public Health level are needed to enhance parental health literacy, currently not standard practice.

